# Turquoise Discoloration of Organs on Autopsy Secondary to Treatment of Septic Shock With Methylene Blue

**DOI:** 10.7759/cureus.10434

**Published:** 2020-09-13

**Authors:** Anoshia Afzal, Michael Quinton, Umar Farooque, Michael Magguilli

**Affiliations:** 1 Pathology, University of Oklahoma Health Sciences Center, Oklahoma City, USA; 2 Neurology, Dow University of Health Sciences, Karachi, PAK

**Keywords:** pseudomonas aeuginosa, autopsy, bacteremia, septic shock, bluish-green discoloration, turquoise discoloration, organs, humans, methylene blue treatment

## Abstract

Septic shock can result from the dissemination of infections and can lead to hypoperfusion secondary to vasodilation. Methylene blue can help stabilize blood pressure refractory to other measures in shock. We report a case of a 58-year-old male who died of septic shock due to *Pseudomonas aeroginosa* bacteremia secondary to acute folliculitis and epididymo-orchitis. He was given methylene blue for reversal of septic shock but he did not respond and expired. Autopsy findings were significant for bluish-green discoloration of organs, especially the heart, lungs, and brain during prosection secondary to methylene blue treatment. It is important to recognize artifacts of treatment and to discern them from changes due to putrefaction or the classic green pigmentation associated with *Pseudomonas aeruginosa *infection, such as chloronychia. The case report illustrates that circulating methylene blue and its metabolites can accumulate in the organs in a dose-related fashion, imparting an interesting turquoise to dark blue-green pigment during the autopsy. Additional studies are warranted to enable pathologists to differentiate among the pigmentation associated with *Pseudomonas aeruginosa* bacteremia, putrefaction, and methylene blue treatment.

## Introduction

Septic shock has a mortality rate of around 20%. It is the most common cause of death in intensive care units (ICU). The outcomes of septic shock vary depending on the extent of the infection and the presence of comorbidities. It is the result of a systemic immune response to infections. In response to the infectious insult, inflammatory mediators cause systemic vasodilatation, manifesting as low blood pressure, leading to tissue hypoperfusion and the vascular congestion seen histologically [1]. Another consequence of sepsis is disseminated intravascular coagulation observed clinically as abnormal laboratory clotting values due to the consumption of clotting factors and the formation of thrombi.

Methylene blue has been used therapeutically dating back to the late 18th century for the treatment of conditions ranging from malaria to carbon monoxide poisoning, and even as a urinary analgesic. Modern use revolves around its mechanism as a reducing agent in the treatment of methemoglobinemia; however, there is also experimental and clinical evidence supporting methylene blue as a treatment for shock refractory to fluid administration, vasoconstrictors, and inotropic agents because it has shown to induce systemic and pulmonary vasoconstriction through its action as a selective inhibitor of guanylate cyclase, a second messenger involved in nitric oxide-mediated vasodilation in patients with septic shock, without a significant decrease in cardiac index [2-4].

## Case presentation

A 58-year-old male with a history of congestive heart failure, hypertension, type II diabetes mellitus, recurrent deep vein thromboses (status post inferior vena cava [IVC] filter placement), pulmonary embolism, and hypothyroidism was admitted with neurologic symptoms in May 2020 related to a T4/T5 mass. Clinical features included bone pain, recurrent infection, spinal cord compression, and peripheral neuropathy. He underwent laminectomy and tumor debulking in June 2020, during which a biopsy of his lesion was taken that revealed a plasmacytoma. Bone marrow biopsy showed no definitive evidence of a monoclonal plasma cell population, but the patient met the criteria for multiple myeloma (plasmacytoma and lytic lesions) and was started on multiple chemotherapeutic agents. After developing pancytopenia and fever post-chemotherapy, he developed septic shock that manifested as lactic acidosis, disseminated intravascular coagulation (DIC), renal failure, and elevated transaminases. He was transferred to the ICU. Blood and scrotal wound cultures were sent that grew *Pseudomonas aeroginosa*. He was intubated and required multiple vasopressors and broad-spectrum antibiotics. Methylene blue was also used to elevate his blood pressure approximately 27 hours prior to death. Due to the poor prognosis, the family chose to pursue comfort care measures only, and the patient expired shortly thereafter due to septic shock.

**Figure 1:**
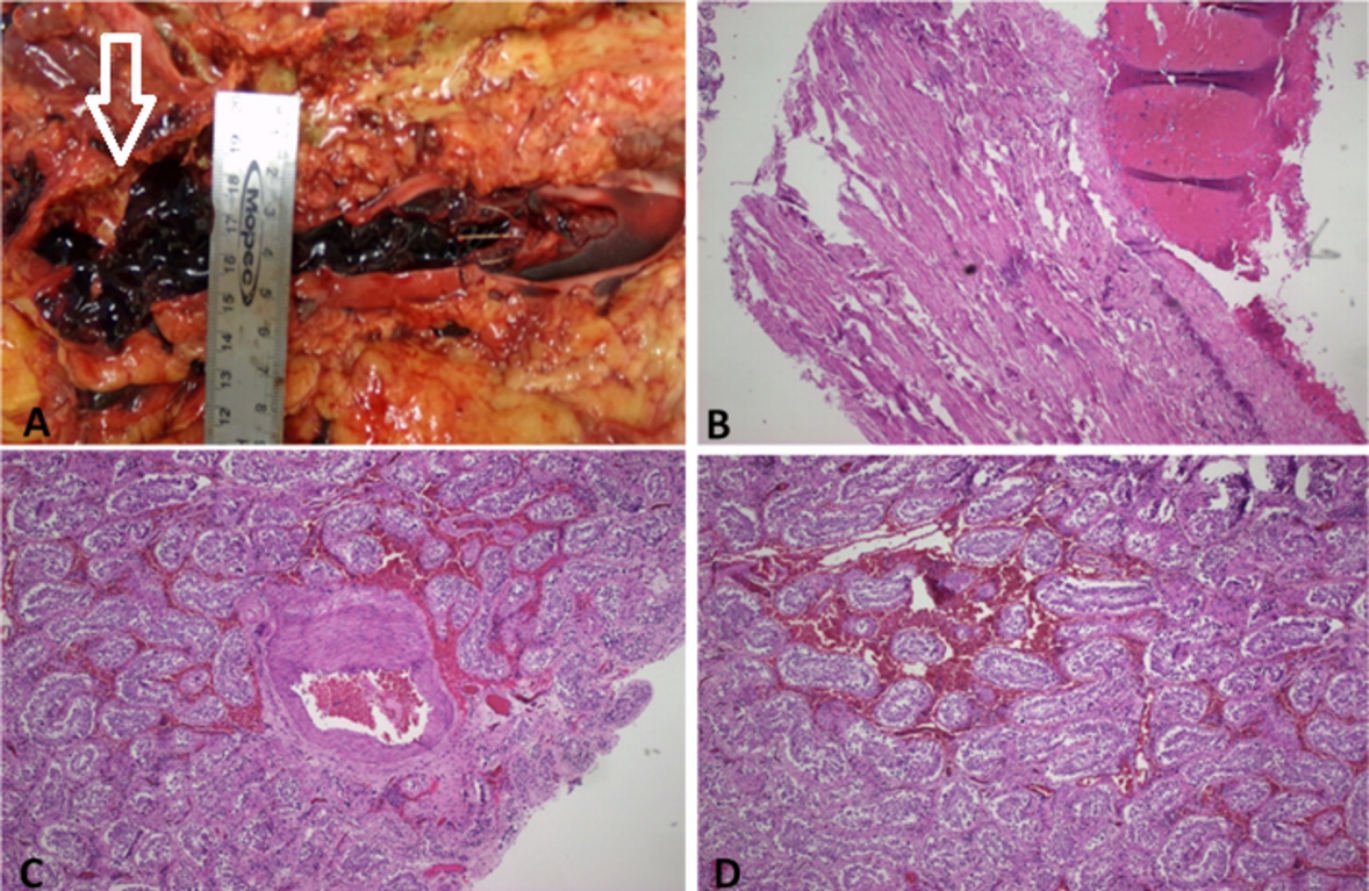
Inferior vena cana (IVC) filter with an entangled clot grossly (A) and microscopically (B). Hemorrhagic (C) and congested (D) left testicle.

An unrestricted autopsy was requested by the family and treating clinicians. The post-mortem examination revealed a full-thickness scrotal ulcer, and an edematous, hemorrhagic left testicle. Gross findings also included superficial sacral decubitus ulcers, severe calcific coronary, and systemic atherosclerotic cardiovascular disease, cardiomegaly, pulmonary edema, bilateral pleural effusions, and thromboemboli entangled in the IVC filter (Figure 1A, 1B). Histologic findings included areas of hemorrhagic necrosis and bacterial overgrowth in the scrotal ulcer sections, hemorrhage (Figure 1C) and congestion (Figure 1D) in the left testicle, and nodular glomerulosclerosis.

**Figure 2:**
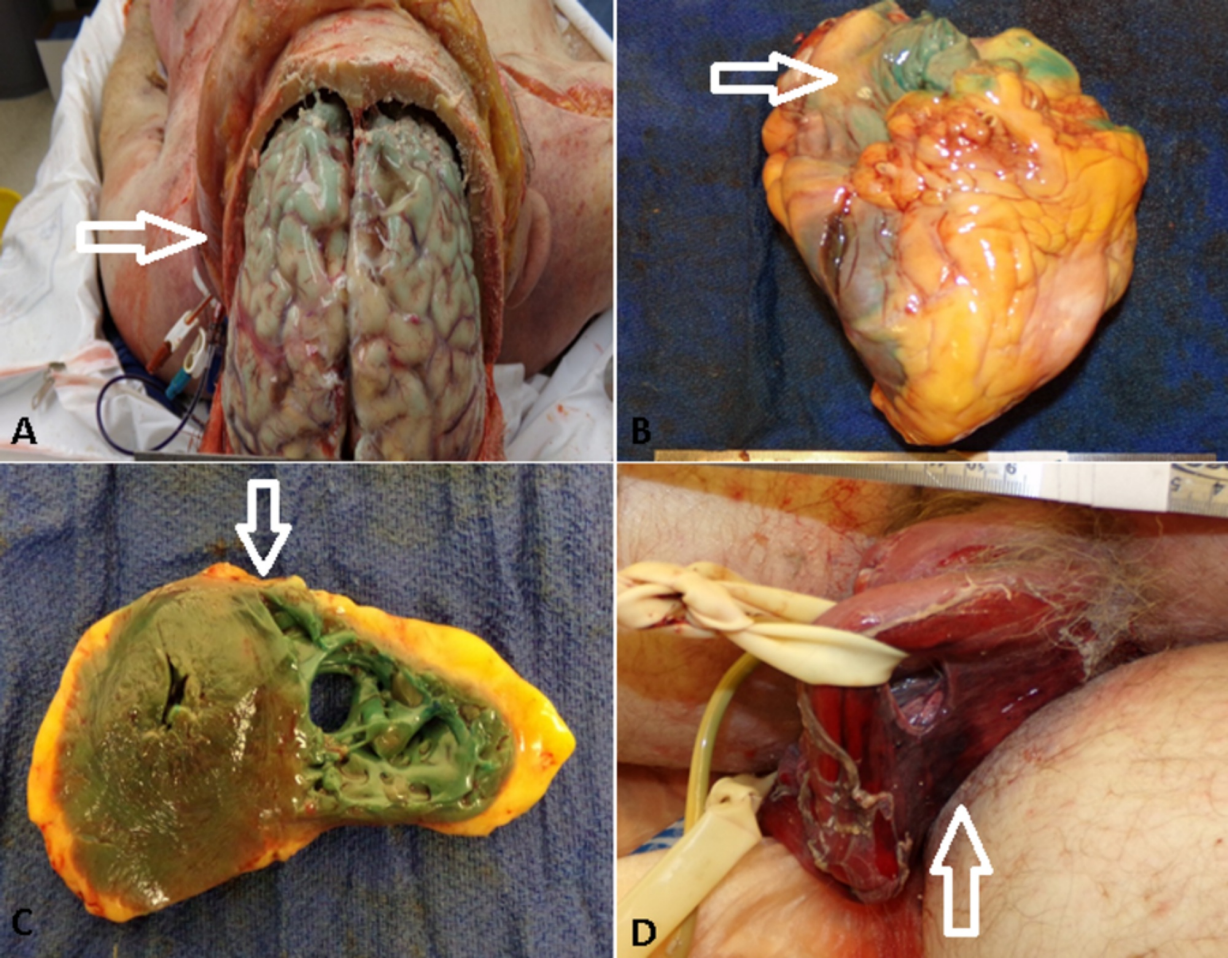
Bluish-green/turquoise discoloration of the brain (A), heart (B), left and right ventricles (C). Perforated scrotal ulcer revealing hemorrhagic left testicle (D).

The unique finding was bluish-green discoloration of organs including the brain (Figure 2A), heart (Figure 2B, 2C), lungs, and kidneys during autopsy, which was secondary to methylene blue treatment in this patient of septic shock due to *Pseudomonas aeroginosa* bacteremia secondary to acute folliculitis and epididymo-orchitis (perforated/ulcerated scrotum with areas of hemorrhagic necrosis) (Figure 2D). This discoloration increased from green to turquoise blue as the organs were set on the table for dissection.

## Discussion

Shock is the result of several potentially lethal clinical entities, including infections, trauma, and cardiovascular collapse. Clinically, shock manifests as systemic hypoperfusion due to either decreased cardiac output, reduced circulating volume, or vasodilation, which results in eventually irreversible tissue injuries in multiple organ systems [1]. Our patient was given methylene blue for septic shock before it was decided not to escalate any further care because of poor prognosis and lack of response to clinical management. An autopsy performed the following day initially revealed a soft, green hue to the heart, lungs, and brain, which intensified to a darker turquoise-green as the prosection proceeded. The circulating methylene blue and its metabolites can accumulate in the organs in a dose-related fashion and can give interesting turquoise to dark blue-green shades during the autopsy. The metabolites azure A and B are responsible for the discoloration along with a colorless metabolite leukomethylene blue which rapidly oxidizes to methylene blue in the presence of oxygen, giving the obvious color change of the myocardium [5-7].

*Pseudomonas aeruginosa* diagnosis depends on laboratory identification and isolation on media. It grows well on most laboratory media and is commonly isolated on blood agar plates or eosin-methylthionine blue agar. It is identified as a gram-negative lactose non-fermenter with a positive oxidase reaction, a fruity odor, and its ability to grow at 42°C [8,9]. It is recognized for producing a blue-green pigmentation in culture growth as well as in infected tissue (chloronychia) secondary to combinations of multiple metabolites, such as pyocyanin and pyoverdine. Pyocyanin is known to retard the growth of some other bacteria which might be a causative factor in facilitating *Pseudomonas aeruginosa *colonization. It can be confusing to differentiate the discoloration caused by two entirely separate etiologies, i.e. methylene blue and* Pseudomonas aeruginosa*.

Our patient was immunocompromised and acquired the infection post-chemotherapy. In short, his multiple myeloma treatment contributed to the septic shock by making him more susceptible to otherwise manageable infections. A plasma cell myeloma is a clonal proliferation of plasma cells or their precursors causing tumor formation, excess immunoglobulin, and osteolytic bone disease. The median survival is three years using standard therapy, and five years with dose-intensive therapy and stem cell transplantation [10].

## Conclusions

Methylene blue accumulation in organs in a dose-related fashion can give interesting turquoise to dark blue-green shades during the autopsy. Therefore, it is essential to identify these features of methylene blue treatment and to distinguish them from changes due to putrefaction or *Pseudomonas aeruginosa *infection associated green pigmentation.
